# Successful application of lorlatinib in a 23‐year‐old patient with anaplastic lymphoma kinase ( *ALK)*‐positive lung cancer and multiple brain metastases

**DOI:** 10.1002/cnr2.1981

**Published:** 2024-01-11

**Authors:** Yosuke Murakami, Yosuke Kawashima, Shinji Chiba, Shuichiro Hara, Yusuke Yamazaki, Tsuyoshi Doman, Shin Saito, Tetsuo Odaka, Takahiro Ogasawara, Hisashi Shimizu, Jun Sugisaka, Tomoiki Aiba, Yukihiro Toi, Shinsuke Yamanda, Yuichiro Kimura, Shunichi Sugawara

**Affiliations:** ^1^ Department of Pulmonary Medicine Sendai Kousei Hospital Miyagi Japan; ^2^ Department of Pulmonary Medicine Iwate Prefectural Central Hospital Iwate Japan

**Keywords:** anaplastic lymphoma kinase *(ALK)*‐positive lung cancer, central nervous system (CNS) metastasis, second‐line treatment

## Abstract

**Background:**

Anaplastic lymphoma kinase (*ALK*)‐positive lung cancer has a better long‐term prognosis with *ALK*‐inhibitor than other lung cancers. However, resistance to *ALK*‐inhibitors and the control of metastases in the central nervous system (CNS) remain to be a challenge in the management of *ALK*‐positive lung cancer.

**Case:**

We present the case of a 23‐year‐old man who developed multiple brain metastases while receiving alectinib treatment for *ALK*‐positive lung cancer. After 3 months of lorlatinib initiation, brain metastases disappeared, and complete response (CR) was maintained.

**Conclusion:**

While lorlatinib can be used as first line therapy, this drug may be considered as second line or later option for patients with multiple brain metastases if the patient has already been treated with other *ALK*‐inhibitors since lorlatinib is thought to have good CNS penetration. This treatment option should be verified by further research.

## 1 INTRODUCTION

Anaplastic lymphoma kinase (*ALK*) is a cancer driver gene mutation found in about 3%–5% of lung cancers.^1^, ^2^, ^3^ One of the characteristics of *ALK*‐positive lung cancer is that it tends to be more common in younger people than other lung cancers, especially the patient with *EML‐4* mutated *ALK* positive lung cancer is reported to be much younger.^4^, ^5^ In the treatment of *ALK*‐positive lung cancer, the choice of *ALK*‐tyrosin kinase inhibitor (TKI) demonstrates a favorable effect though the majority of cases fall into the acquisition of resistance to the *ALK*‐TKI. Moreover, it is reported that ALK‐positive lung cancer patients tend to relapse with central nervous system (CNS) metastasis.^6^

Lorlatinib is a third‐generation *ALK‐*TKI with favorable CNS penetration and it is considered as an option in the treatment of *ALK*‐positive lung cancer with CNS metastasis.^7^ There is a phase II study for lorlatinib in patients with CNS‐only progression on second‐generation *ALK*–inhibitors.^8^ In the study, 15 of 23 (65%) patients had irradiated CNS metastases, with a median of 20.2 months between radiation and lorlatinib. Conversely, there are limited data about the efficacy of lorlatinib against radiation naïve patients due to the data from a small number of cases. Here we report a case wherein lorlatinib demonstrated significant and rapid improvement of symptomatic brain metastases in a patient with no prior radiation therapy.

## 2 CASE PRESENTATION

Case: a 23‐year‐old man.

Medical History: no medical history.

**FIGURE 1:**
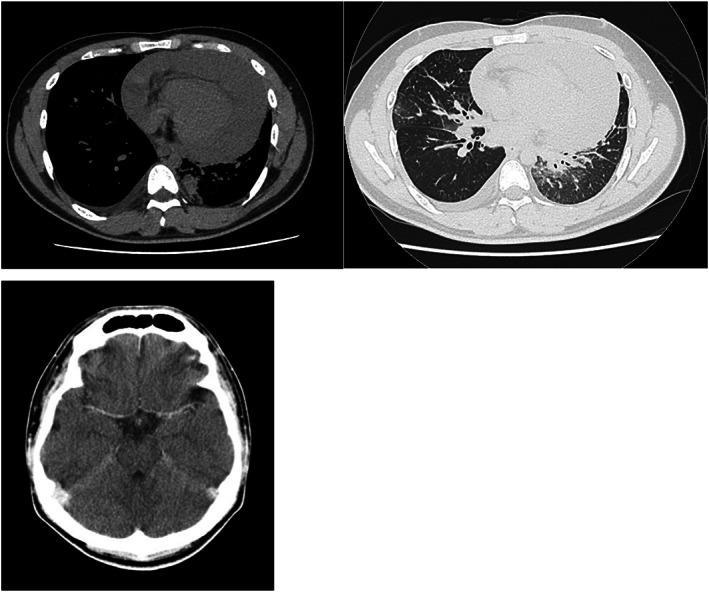
Initial computed tomography (CT) scan showing pericardial effusion and multiple granular shadows and consolidations in both lungs with no evidence of central nervous system metastasis.

Diagnostic process: On March 23, 2022, he visited a hospital (Iwate Prefectural Central Hospital, Iwate, Japan) for chest and abdominal pain. Computed tomography (CT) revealed multiple intrapulmonary nodules and masses, bilateral pleural effusions, pericardial effusions, and multiple mediastinal lymph nodes. Contrast‐enhanced CT scan of the brain showed no metastases to the central nervous system (Figure 1). Pericardial drainage was performed, and cytology of the pericardial fluid confirmed the histology of adenocarcinoma. Thereafter ultrasound‐guided transbronchial needle aspiration of mediastinum lymph node was performed. The lymph node was pathologically confirmed as metastasis of lung adenocarcinoma and Oncomine Dx Target Test revealed that the tumor was *ALK*‐positive. Based on these findings, the patient was diagnosed with *ALK*‐positive lung adenocarcinoma (cTXN3M1c: stage IVB).

**FIGURE 2:**
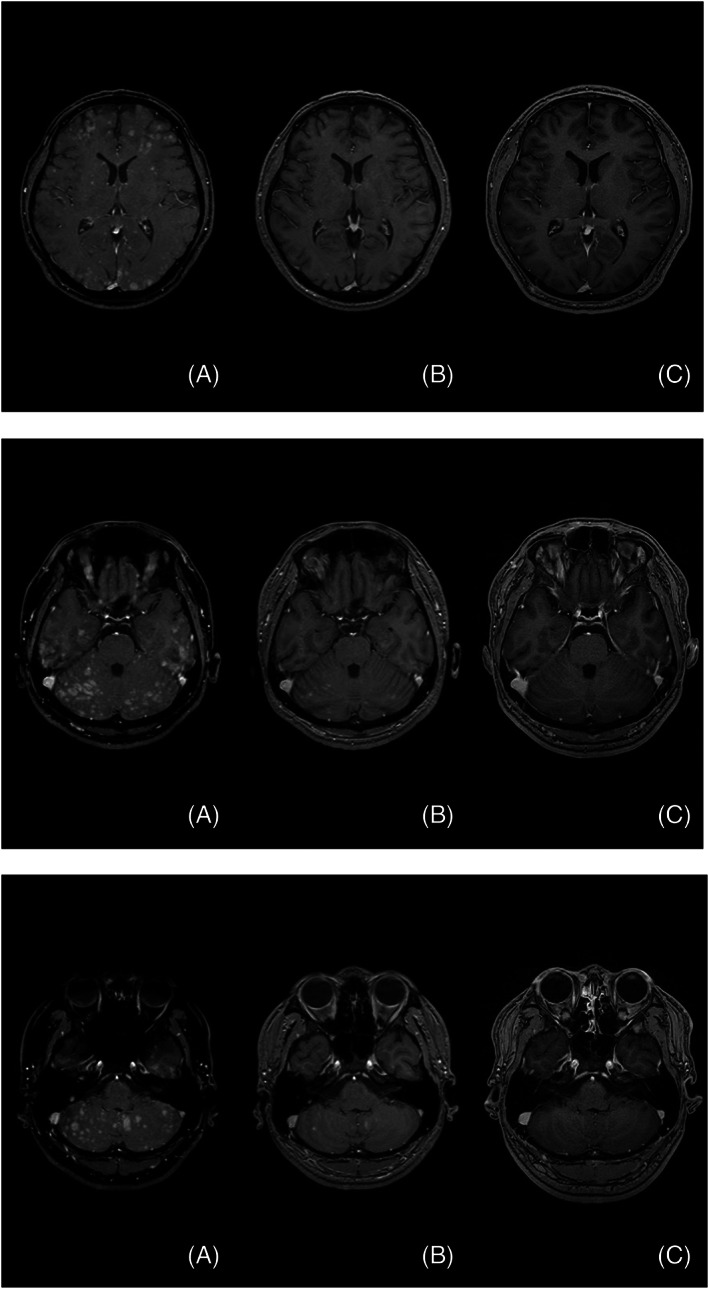
Contrast‐enhanced magnetic resonance imaging (MRI) scan of the head. Before lorlatinib administration, multiple brain metastases were observed (A); however, 12 days after lorlatinib administration, many of the metastases had disappeared (B), and after three months, multiple brain metastases were no longer detected on MRI (C).

Treatment: From April 28, 2022, the patient was started on alectinib (300 mg/day) as first‐line therapy. The multiple mediastinal lymph nodes reduced in size (partial response) and bilateral pleural effusion and pericardial effusion also decreased. There were no adverse events associated with the use of alectinib. However, 8 months after alectinib initiation, the patient developed symptoms of headache and vomiting. Contrast‐enhanced magnetic resonance imaging (MRI) of the brain showed multiple brain metastases (Figure 2). After consultation with the patient, treatment with lorlatinib (100 mg/day) was initiated as second‐line therapy from January 20, 2023. Only 12 days after starting lorlatinib, contrast‐enhanced MRI of the brain showed that multiple brain metastases had generally decreased in size (Figure 2) and his symptoms had dramatically improved. Adverse events associated with the use of lorlatinib were grade 1 elevated total cholesterol and grade 2 elevated triglycerides defined by Common Terminology Criteria for Adverse Events (CTCAE), both of which were manageable with the use of a statin. After 3 months of treatment with lorlatinib, the patient remains in good condition and contrast‐enhanced MRI of the brain showed that multiple brain metastases had completely disappeared (Figure 2).

Follow‐up: The response had been maintained continuously for 6 months, when new brain metastases were found in MRI on July 25, 2023.

## 3 DISCUSSION

CNS metastases occur in 20%–70% of patients with *ALK*‐positive lung cancer, and CNS recurrence is frequent during treatment with *ALK* inhibitors.^6^ If the site of exacerbation is limited to brain, radiotherapy is one of the options. However, whole brain radiation therapy should be carefully considered because late effects of whole brain radiation therapy include decreased quality of life and cognitive decline, which can be a serious problem, especially for younger patients.^9^

Lorlatinib is effective against CNS metastases because of its good permeability of the blood–brain barrier and its protective effect on neurons.^10^ Lorlatinib may be effective as an *ALK* inhibitor after first‐line therapy because it shows antitumor activity against multiple *ALK* mutants, including acquired mutations that confer resistance to other *ALK* inhibitors.^7^

As far as we could find, there are two case reports of lorlatinib for CNS metastasis after treatment with other *ALK* inhibitors, both of which showed resolution of metastasis and no serious adverse events, suggesting that lorlatinib is effective in the treatment of CNS metastasis.^11^, ^12^ Although previous study reported that brain metastasis is one of risk factors of CNS adverse events of lorlatinib, these adverse events can often be controlled by drug withdrawal and dose reductions.^13^ Thus, lorlatinib is a treatment option for *ALK*‐positive lung cancer with multiple brain metastases or meningeal dissemination after first‐line therapy.

There are two notable points in this case compared with previous reports. First, especially in young patients such as in this case, late side effects of whole brain radiation therapy is of significant concern and should be avoided whenever possible. This case demonstrated rapid CNS response to lorlatinib without serious adverse events, suggesting that in some cases, lorlatinib could be offered prior to whole brain radiation. Second, the brain metastases were generally decreased only after 12 days after the initiation of lorlatinib, suggesting that the effect of lorlatinib on the CNS might be rapid. This might reflect that loratinib is rapidly absorbed with peak plasma concentrations occurring 1–2 h after dosing.^14^ In addition, loratinib cerebrospinal fluid concentrations reach over 70% of loratinib free‐plasma concentrations.^15^

## 4 CONCLUSION

We report a clinically suggestive case who was especially young and multiple brain metastases during alectinib treatment were treated by lorlatinib. We believe that the patient could avoid immediate whole brain radiation therapy, which is a treatment with concerns about side effects including late effects. In clinical setting, we often encounter cases like this and should select an appropriate therapy depending on the patient's condition. Although there is a phase II study for lorlatinib in patients with CNS‐only progression on second‐generation *ALK*–inhibitors, it is limited data due to a small number. Therefore, lorlatinib treatment for the patient population like this case should be verified by further research.

## AUTHOR CONTRIBUTIONS

**Yosuke Murakami:** Conceptualization (lead); writing – original draft (lead). **Yosuke Kawashima:** Conceptualization (supporting); writing – review and editing (supporting). **Shinji Chiba:** Conceptualization (supporting); writing – review and editing (supporting). **Shuichiro Hara:** Conceptualization (supporting); writing – review and editing (supporting). **Yusuke Yamazaki:** Conceptualization (supporting); writing – review and editing (supporting). **Tsuyoshi Doman:** Conceptualization (supporting); writing – review and editing (supporting). **Shin Saito:** Conceptualization (supporting); writing – review and editing (supporting). **Tetsuo Odaka:** Conceptualization (supporting); writing – review and editing (supporting). **Takahiro Ogasawara:** Conceptualization (supporting); writing – review and editing (supporting). **Hisashi Shimizu:** Conceptualization (supporting); writing – review and editing (supporting). **Jun Sugisaka:** Conceptualization (supporting); writing – review and editing (supporting). **Tomoiki Aiba:** Conceptualization (supporting); writing – review and editing (supporting). **Yukihiro Toi:** Conceptualization (supporting); writing – review and editing (supporting). **Shinsuke Yamanda:** Conceptualization (supporting); writing – review and editing (supporting). **Yuichiro Kimura:** Conceptualization (supporting); writing – review and editing (supporting). **Shunichi Sugawara:** Conceptualization (supporting); writing – review and editing (supporting).

## CONFLICT OF INTEREST STATEMENT

Yosuke Kawashima received personal fees from Taiho Pharmaceutical, Eli Lilly, Life Technologies Japan Ltd, Chugai Pharma, AstraZeneca, and Kyowa Kirin. Yukihiro Toi received personal fees from AstraZeneca, Chugai Pharma, Pfizer, Taiho Pharmaceutical, Kyowa Kirin, Bristol‐Myers Squibb, Ono Pharmaceutical, and MSD K.K. Shinsuke Yamanda received personal fees from AstraZeneca, Novartis, Sanofi, GSK, and Nippon Boehringer Ingelheim. Shunichi Sugawara received personal fees from AstraZeneca, Chugai Pharma, Pfizer, Taiho Pharmaceutical, Eli Lilly, Novartis, Kyowa Kirin, Bristol‐Myers Squibb, Ono Pharmaceutical, MSD K.K and Nippon Boehringer Ingelheim. All other authors declare no conflict of interest.

## ETHICS STATEMENT

None required.

## INFORMED CONSENT

Written informed consent was obtained from the patient for publishing this case report and accompanying images.

## Data Availability

Data sharing is not applicable to this article as no new data were created or analyzed in this study.
